# Propelling sustainable energy: Multi-omics analysis of pennycress *FATTY ACID ELONGATION1* knockout for biofuel production

**DOI:** 10.1093/plphys/kiae650

**Published:** 2024-12-09

**Authors:** Amira Rasoul, Christopher R Johnston, Jordan LaChance, John C Sedbrook, Ana Paula Alonso

**Affiliations:** Department of Biological Sciences and BioDiscovery Institute, University of North Texas, Denton, TX 76203, USA; Department of Biological Sciences and BioDiscovery Institute, University of North Texas, Denton, TX 76203, USA; Department of Biological Sciences and BioDiscovery Institute, University of North Texas, Denton, TX 76203, USA; School of Biological Sciences, Illinois State University, Normal, IL 61790, USA; Department of Biological Sciences and BioDiscovery Institute, University of North Texas, Denton, TX 76203, USA

## Abstract

The aviation industry’s growing interest in renewable jet fuel has encouraged the exploration of alternative oilseed crops. Replacing traditional fossil fuels with a sustainable, domestically sourced crop can substantially reduce carbon emissions, thus mitigating global climate instability. Pennycress (*Thlaspi arvense L*.) is an emerging oilseed intermediate crop that can be grown during the offseason between maize (*Zea mays*) and soybean (*Glycine max*) to produce renewable biofuel. Pennycress is being domesticated through breeding and mutagenesis, providing opportunities for trait enhancement. Here, we employed metabolic engineering strategies to improve seed oil composition and bolster the plant's economic competitiveness. *FATTY ACID ELONGATION1* (*FAE1*) was targeted using CRISPR-Cas 9 gene editing to eliminate very long chain fatty acids (VLCFAs) from pennycress seed oil, thereby enhancing its cold flow properties. Through an integrated multiomics approach, we investigated the impact of eliminating VLCFAs in developing and mature plant embryos. Our findings revealed improved cold-germination efficiency in *fae1*, with seedling emergence occurring up to 3 d earlier at 10 °C. However, these alterations led to a tradeoff between storage oil content and composition. Additionally, these shifts in lipid biosynthesis were accompanied by broad metabolic changes, such as the accumulation of glucose and ADP-glucose quantities consistent with increased starch production. Furthermore, shifts to shorter FA chains triggered the upregulation of heat shock proteins, underscoring the importance of VLCFAs in stress signaling pathways. Overall, this research provides crucial insights for optimizing pennycress seed oil while preserving essential traits for biofuel applications.

## Teaser

Pennycress *FATTY ACID ELONGATION1* knockout leads to elimination of very long chain fatty acids, better cold-germination efficiency, central metabolic shifts, and upregulation of stress-related genes.

## Introduction

Air travel is a vital cornerstone of modern society, but comes with environmental challenges due to the ever-growing demand, rise in energy consumption, and subsequent carbon emissions (Kousoulidou and Lonza 2016; Doliente et al. 2020). To address this, federal agencies are exploring sustainable aviation fuels (SAFs) from alternative crops like pennycress (*Thlaspi arvense*), as a means to reduce carbon emissions (Trejo-Pech et al. 2019; Arias et al. 2023). Pennycress, a member of the Brassicaceae family, exhibits a distinctive array of characteristics that make it both a sustainable and economically favorable choice for aviation fuel production (Chopra et al. 2020; Phippen et al. 2022). It thrives worldwide in temperate regions with minimal nutritional demands and displays exceptional cold-tolerance allowing it to be integrated into a double cropping system (Moore et al. 2022). This agricultural system is well suited to fill the fallow period between maize (*Zea mays*) and soybean (*Glycine max*) cultivations in the US Midwest, effectively safeguarding the otherwise barren land from soil erosion and nutrient depletion (Phippen and Phippen 2012; Fan et al. 2013; Johnson et al. 2015; Moore et al. 2020). Furthermore, mature pennycress seeds contain a relatively high content of oil, ranging from 13.5% to 38.7% oil by dry weight (DW), outperforming traditional oilseed crops in technoeconomic assessments (Sedbrook et al. 2014; Trejo-Pech et al. 2019). Collectively, these attributes underscore the potential of pennycress as an ideal candidate for biofuel production.

Seed oil reserves, while undoubtedly a valuable resource for SAF production, serve the primary function of supporting the growth and development of germinating seedlings. Immature embryos must generate adequate energy reserves to provide for the germinating seedling before it can photosynthesize. Carbon and nitrogen precursors are converted into reserves, such as seed oils, proteins, and carbohydrates, via central metabolic pathways (Goffman et al. 2005). During embryonic development, de novo fatty acid synthesis (FAS) gives rise to long chain FAs (chain length ≤18 carbons). Subsequently, the fatty acid elongation (FAE) complex, localized at the endoplasmic reticulum (ER), facilitates the production of very long chain fatty acids (VLCFAs) or FAs with a chain length ≥20. These processes require energy, reductant, and a carbon source provided by central carbon metabolism to proceed (Pleite et al. 2005; Sagun et al. 2023). In photosynthetic embryos, like those found in pennycress, a portion of the requisite energy and reducing power can be sourced from photosynthesis (Goffman et al. 2005; Allen et al. 2009; Arias et al. 2023). Other pathways such as the oxidative pentose phosphate pathway (OPPP), oxidative phosphorylation, glycolysis, and the tricarboxylic acid (TCA) cycle play integral roles in the generation of energy and reductant (Tsogtbaatar et al. 2015; Tsogtbaatar et al. 2020; Johnston et al. 2022). A comprehensive understanding of these metabolic pathways and how they contribute to oil biosynthesis is a key aspect of crop improvement.

The carbon source for FAS originates from the parent plant. Parentally provided sucrose is enzymatically cleaved by invertase into products that enter glycolysis and/or the OPPP. Subsequently, pyruvate generated by these pathways is converted into acetyl-CoA by the pyruvate dehydrogenase complex in the plastid to be readily available for de novo FAS. During de novo, FAS 2 carbons are added cyclically until a 16:0-ACP is generated, which may then be further elongated by 3-ketoacyl-ACP synthase II to generate 18:0-ACP. 18:0-ACP can then be desaturated by stearoyl-ACP Δ9-desaturase introducing a double bond at the 9th carbon position (Sagun et al. 2023). Following de novo FAS, the ACP-acyl group is cleaved into a free FA and ACP by fatty acyl-ACP thioesterases in the inner envelope of the plastid. The free FA then crosses through the outer plastid envelope membrane to the cytosol via the suggested protein FA export 1 where long chain acyl-coenzyme A activates it to the CoA thioester prior to its export to the ER for elongation (Hölzl and Dörmann 2019).

The primary carbon source for FAE, citrate, originates from the TCA cycle. Citrate is transported to the cytosol where it then undergoes conversion into oxaloacetate and acetyl-CoA by ATP dependent citrate lyase. Acetyl-CoA is then further transformed into malonyl-CoA by homomeric ACCase for the initial step of the elongation process. The FAE complex includes 4 ER membrane bound enzymes. Elongation starts with the condensation of malonyl-CoA with a long chain acyl-CoA by 3-ketoacyl-CoA synthase (KCS) utilizing ATP to generate 3-ketoacyl-CoA followed by a reduction to 3-hydroxyacyl-CoA by 3-ketoacyl-CoA reductase using NAD(P)H. 3-Hydroxyacyl-CoA then undergoes a dehydration to form enoyl-CoA by 3-hydroxyacyl-CoA dehydratase. Lastly, there is a final reduction of enoyl-CoA by trans-2,3-enoyl CoA reductase using NAD(P)H resulting in an elongated acyl-CoA. Two carbons are added sequentially per round until the intended length is achieved. Beginning at ATP citrate lyase, FAE requires a total of 3 energy and 2 reductant equivalents per round (Haslam and Kunst 2013).

Enhancing seed oil content and composition in pennycress embryos is crucial for its widespread adoption as an intermediate oilseed crop for biofuel production (Sedbrook et al. 2014). Pennycress, being highly amenable to genetic modification and closely related to *Arabidopsis thaliana*, allows for the application of metabolic engineering strategies developed for *Arabidopsis* to be applied in pennycress (Chopra et al. 2020). Targeting genes to reduce VLCFAs in pennycress is important as high VLCFA content can hinder certain biofuel properties, leading to issues like filter clogging at low temperatures (Mofijur et al. 2017). To target VLCFA content *FATTY ACID ELONGATION1* (*FAE1*) loss-of-function mutants were generated through CRISPR-Cas9 gene editing (McGinn et al. 2019). *FAE1* encodes the condensing enzyme 3-ketoacyl-CoA synthase 18 (KCS18) responsible for the initial rate limiting step of FAE (Yan et al. 2015). KCS18 is a member of a broader KCS family including 21 condensing enzymes. The KCS family encompasses functionally redundant condensing enzymes that catalyze the initial rate limiting step of FAE. Each KCS enzyme demonstrates unique substrate specificity determined by the carbon chain length of the initial acyl-CoA substrate (Joubès et al. 2008). Additionally, their expression is tissue specific, for instance *FAE1* encoding KCS18 is only expressed in developing embryos. This characteristic is important as any modifications to oil composition would be embryo-specific.

The genetic modification of *FAE1* is hypothesized to disrupt central carbon metabolism, particularly pathways contributing reductant, energy, and carbon precursors for FAE. Furthermore, a shift in FA composition is predicted to cause alterations in downstream lipid macromolecules such as phospholipids and sphingolipids impacting membrane composition and cellular signaling (Supplementary Fig. S1). Utilizing an integrated approach, we investigated the impact of eliminating VLCFAs in developing plant embryos by examining key aspects such as seed germination, biomass accumulation, metabolite quantities, and gene expression profiles. This work offers a unique perspective by examining *fae1* mutants across key developmental stages, thereby uncovering critical metabolic and transcriptional dynamics during seed filling that have been previously overlooked in studies exclusively examining mature embryo traits (Ozseyhan et al. 2018; Batsale et al. 2021; Jarvis et al. 2021). Notably, this study presents a multiomics analysis of *FAE1*, providing a comprehensive understanding of the role of VLCFAs in developing plant embryos. These findings bridge gaps in the existing literature and offers crucial insight for optimizing pennycress seed oil composition for biofuel applications while retaining essential agronomic traits.

## Results

### *FAE1* mutation alters FA profile in mature embryos influencing seed germination

Pennycress mutant alleles were generated using CRISPR-Cas9 genome editing, targeting *FAE1* as previously described, to enhance seed oil composition for fuel, feed, and food applications (McGinn et al. 2019). The expression of *FAE1* is embryo-specific suggesting that the modification of this gene would have a minimal impact on other plant tissues. To test this, an analysis was conducted to assess several variables such as plant height, leaf count, and the photosynthetic capacity of both leaves and silicles. The results showed minimal differences in plant morphology and physiology between wild-type (WT) plants and the *fatty acid elongation 1*-3 (*fae1-3*) and *fatty acid elongation 1-4* (*fae1-4*) knockout mutant alleles. While at times, there were slight differences in growth between the mutants and WT, the final heights, numbers of leaves, and photosynthetic capacities were essentially the same (Supplementary Fig. S2). These results indicate that the genetic modification of *FAE1*, targeting seed oil composition, does not adversely impact nonembryonic plant growth and development.

**Figure 1.**
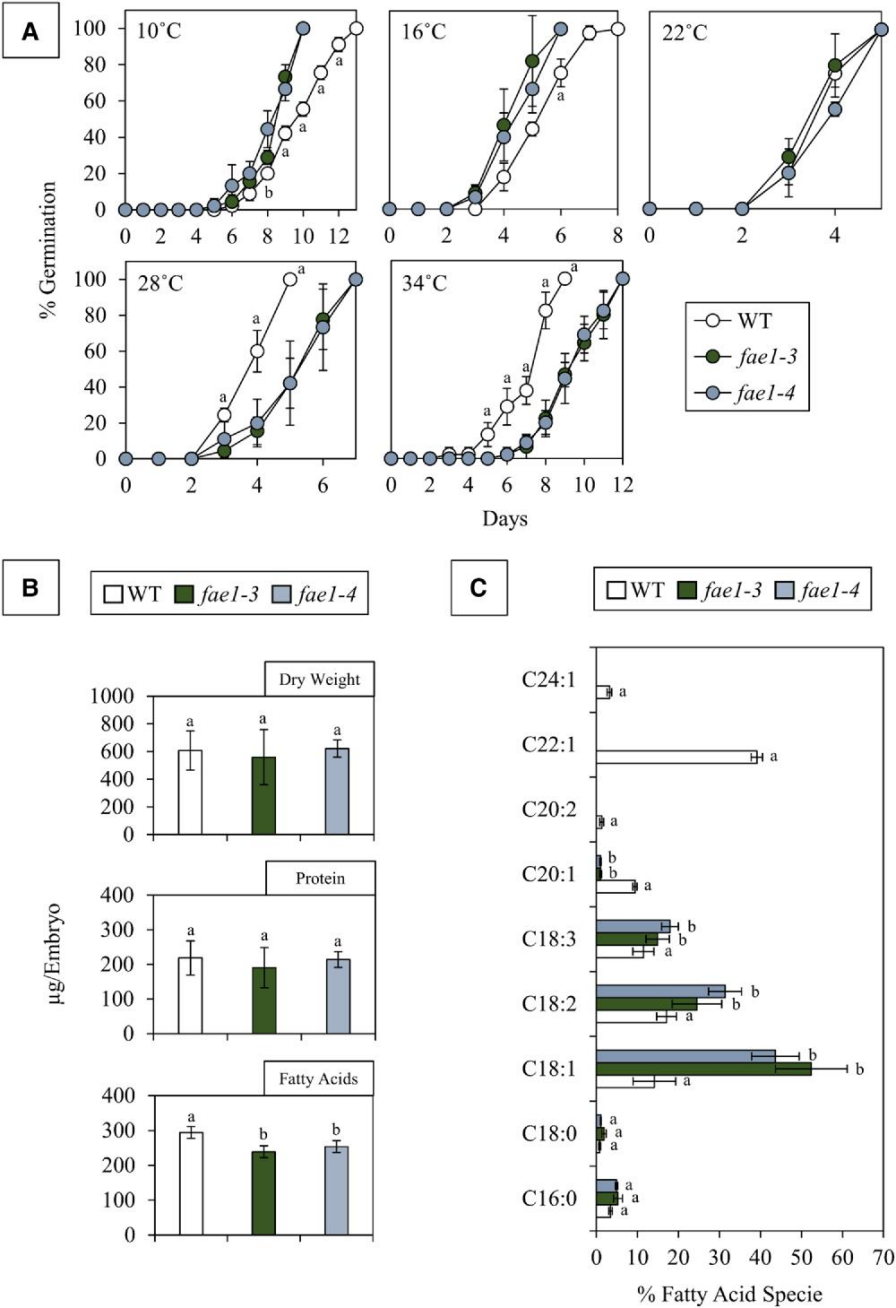
Germination efficiency and mature embryo biomass. **A)** Germination efficiency comparison between WT, *fae1-3*, and *fae1-4* across 5 different temperatures. As determined by an ANOVA, “a” indicates a significant difference between the WT and both *fae1* alleles, while “b” denotes significant differences between the WT and a single *fae1* allele (*P* ≤ 0.05), *n* = 3. **B)** Biomass comparison between WT, *fae1-3*, and *fae1-4* at maturity reveals the amount (μg per embryo) of DW, total FAs, and protein. **C)** FA composition comparison in mature WT, *fae1-3*, and *fae1-4* embryos displays the FA specie in the nomenclature Ca:b where “a” indicates the number of carbons while “b” indicates the number of double bonds. for **B** and **C)** an ANOVA was conducted to examine the differences in among 3 distinct groups. Letters “a” and “b” are utilized to denote significant differences between groups (*P* ≤ 0.05), *n* = 10. Bars represent the Sd of the data across biological replicates.

The biomass content and composition of mature pennycress embryos was assessed to determine potential differences in DW, protein content, and FA content and composition between the WT and *fae1* alleles. In mature pennycress embryos, the average DW and protein content on a per embryo basis was not significantly different between the WT and the *fae1* alleles (Fig. 1B). The WT exhibited a higher total FA content than the *fae1* alleles with an average of 295 ± 24 µg/embryo compared with *fae1-3* and *fae1-4*, which contained 239 ± 23 (*P*-value = 0.00003) and 254 ± 22 (*P*-value = 0.00127) µg/embryo, respectively (Supplementary Table S1). However, it is important to note, mole-based analysis revealed that while the FA content differs when expressed in mass (µg/embryo), this is not observed when expressed in moles (nmol/embryo), indicating that the observed changes in FA quantity (µg/embryo) is likely a reflection of shifts in FA chain length and molecular weight (Supplementary Fig. S3). Indeed, all FA species were significantly different between the WT and both *fae1* alleles, except for stearic acid (C18:0), which only displayed significant differences between the WT and *fae1-3* (Fig. 1C). The most striking modifications identified in the *fae1* alleles was a 3-fold increase in oleic acid (C18:1; 52% and 44% in *fae1-3* and *fae1-4*, respectively, vs. 14% in WT) and the absence of erucic acid (C22:1; 39% in WT).

Given the observed differences in FA composition in mature embryos, experiments were conducted to test germination efficiency at different temperatures (Fig. 1A; Supplementary Table S2). For the temperature-dependent germination experiment, 5 different temperatures ranging from 10 to 34 °C were tested. At 10 °C, both *fae1* alleles displayed significant differences in germination efficiency from day 8 to 12 compared with WT; the mutant alleles exhibited an advantage, reaching 100% germination an average of 3 d sooner. This pattern was also observed at 16 °C; however, the disparity was less pronounced, noting a significant difference only at day 6. At optimal physiological temperatures based on the literature (22 °C) no notable differences were observed, both lines reaching 100% germination within the same timeframe of 5 d. At warmer temperatures (28 °C) germination in *fae1-3* and *fae1-4* was delayed by an average of 2 d. Similarly, at 34 °C, the *fae1* alleles germinated an average 3 d later than the WT. Overall, the mutants tested germinated more rapidly at cooler temperatures and slower at warmer temperatures.

### Developing *fae1-3* embryos exhibit elevated oleic acid and starch content during seed filling

**Figure 2.**
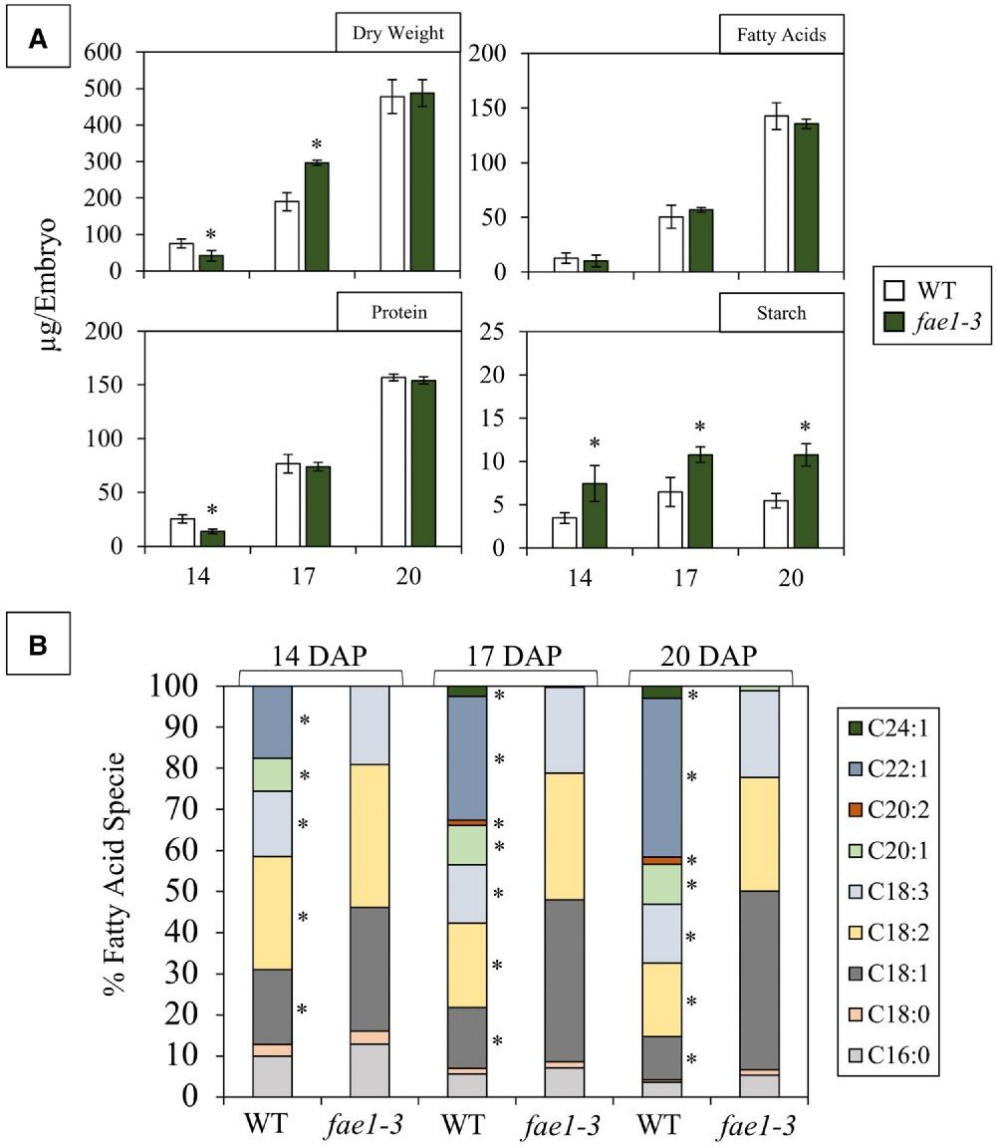
Developing embryo biomass. **A)** Biomass comparison between WT and *fae1-3* at 14, 17, and 20 DAP embryos reveals the amount (μg per embryo) of DW, total FAs, protein, and starch. **B)** FA composition comparison in developing embryos displays the FA specie in the nomenclature Ca:b where “a” indicates the number of carbons while “b” indicates the number of double bonds. Bars represent the Sd of the data across biological replicates. for **A** and **B)** a *t*-test was applied to assess the differences between groups. Asterisks indicate significance (*P* ≤ 0.05), *n* = 4.

To better understand the observed variation in carbon partitioning between mature WT and *fae1-3* embryos, biomass analyses were conducted in developing embryos at 3 developmental stages (14, 17, and 20 d after pollination [DAP]). These stages were selected as they correspond with linear biomass accumulation (Tsogtbaatar et al. 2015; Johnston et al. 2022). During the process of seed filling, the average FA content per embryo was not significantly different between the WT and *fae1-3* (Fig. 2A). Despite the comparable FA content, a notable shift was observed in the FA composition in developing embryos, wherein the absence of VLCFA synthesis contributed to the accumulation of long chain monounsaturated and polyunsaturated FAs such as oleic (C18:1), linoleic (C18:2), and linolenic acids (C18:3) (Fig. 2B). Saturated long chain FAs such as palmitic (C16:0) and stearic acids (C18:0) were not significantly impacted. Furthermore, when examining starch content per embryo, it was found to be ∼2 times higher in *fae1-3* embryos at each developmental stage (Supplementary Table S3). Lastly, a small degree of variation was seen in embryo DW and protein content per embryo between the WT and *fae1* alleles during early development; however, no discernable trend emerged. Differences in starch content and FA composition prompted further investigation into the underlying mechanisms and metabolism behind carbon partitioning and utilization in developing pennycress embryos.

### Targeted metabolic analysis reveals distinct differences in central metabolism in *fae1-3* developing embryos

**Figure 3.**
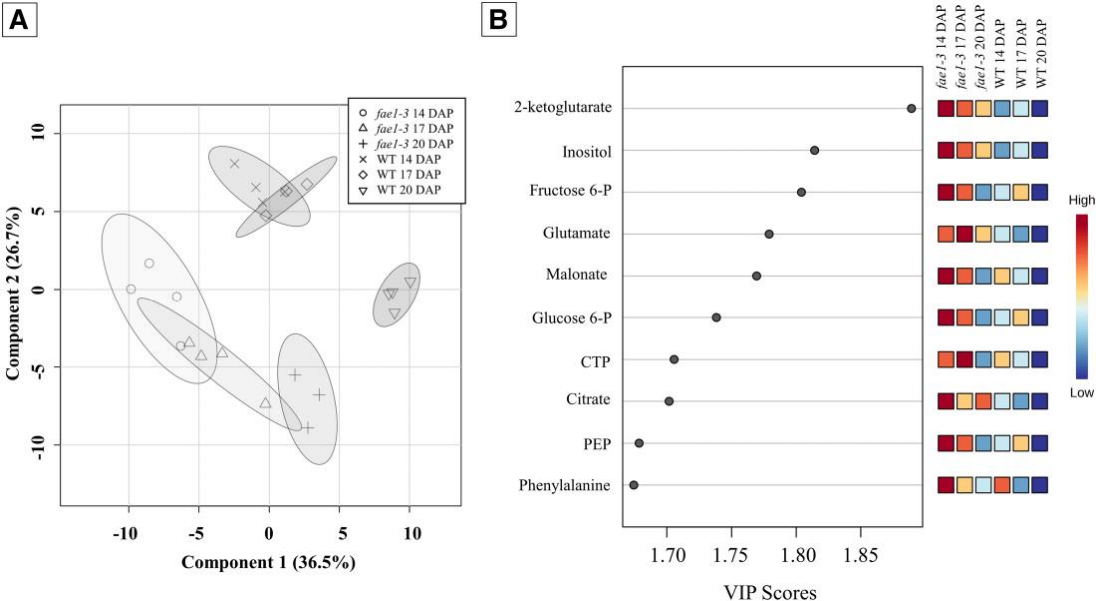
Metabolic profiles of developing embryos. **A)** PLS-DA using metabolomic dataset. PLS-DA was carried out on log-transformed and auto-scaled (i.e. mean-centered and divided by the Sd of each feature) metabolite concentrations expressed in pmol mg DW^−1^ using MetaboAnalyst. The shaded region indicated the 95% CI. The letter “×,” diamond, and upside-down triangle signify the 3 stages, 14, 17, and 20 DAP, respectively in the WT. In *fae1-3* a circle, triangle, and plus sign signify 14, 17, and 20 DAP, respectively. **B)** VIP score plot for the top 10 most important metabolite features contributing the separation identified by PLS-DA. *n* = 4.

To investigate the underlying pathways contributing to the observed differences in biomass profiles between the WT and *fae1-3*, intracellular metabolites were extracted and quantified as previously described using a targeted metabolomics approach (Alonso et al. 2010; Cocuron et al. 2014; Cocuron and Alonso 2014; Johnston et al. 2022). One hundred and seven metabolites involved in primary metabolic pathways, including amino acids, sugars, sugar alcohols, organic acids, and phosphorylated compounds, were quantified at 3 distinct developmental stages, namely, 14, 17, and 20 DAP (Supplementary Table S4). To discern metabolic changes occurring during pennycress embryo development, statistical analyses were performed on the entire metabolomics data set. Partial least squares discriminant analysis (PLS-DA) displayed metabolome segregation between the WT and *fae1-3*, as well as between developmental time points (Fig. 3A). Component 1 accounted for 36.5% of the separation, while component 2 described 26.7% of the observed separation, resulting in a total variance of 63.2%. The importance of the variables contributing to the separation projected by the PLS-DA was calculated, and the top 10 contributing metabolites are shown in the variable importance in projection (VIP) score plot (Fig. 3B). These important metabolites found at higher levels in *fae1-3* include glycolytic and TCA cycle intermediaries such as glucose 6-phosphate, fructose 6-phosphate, phosphoenolpyruvate, citrate, and 2-ketoglutarate respectively, amino acids phenylalanine and glutamate, and malonate. These important metabolites, particularly malonate, due to its proximity to the initial rate limiting step of FAE, underscore the importance of evaluating metabolic shifts in *fae1-3*.

**Figure 4.**
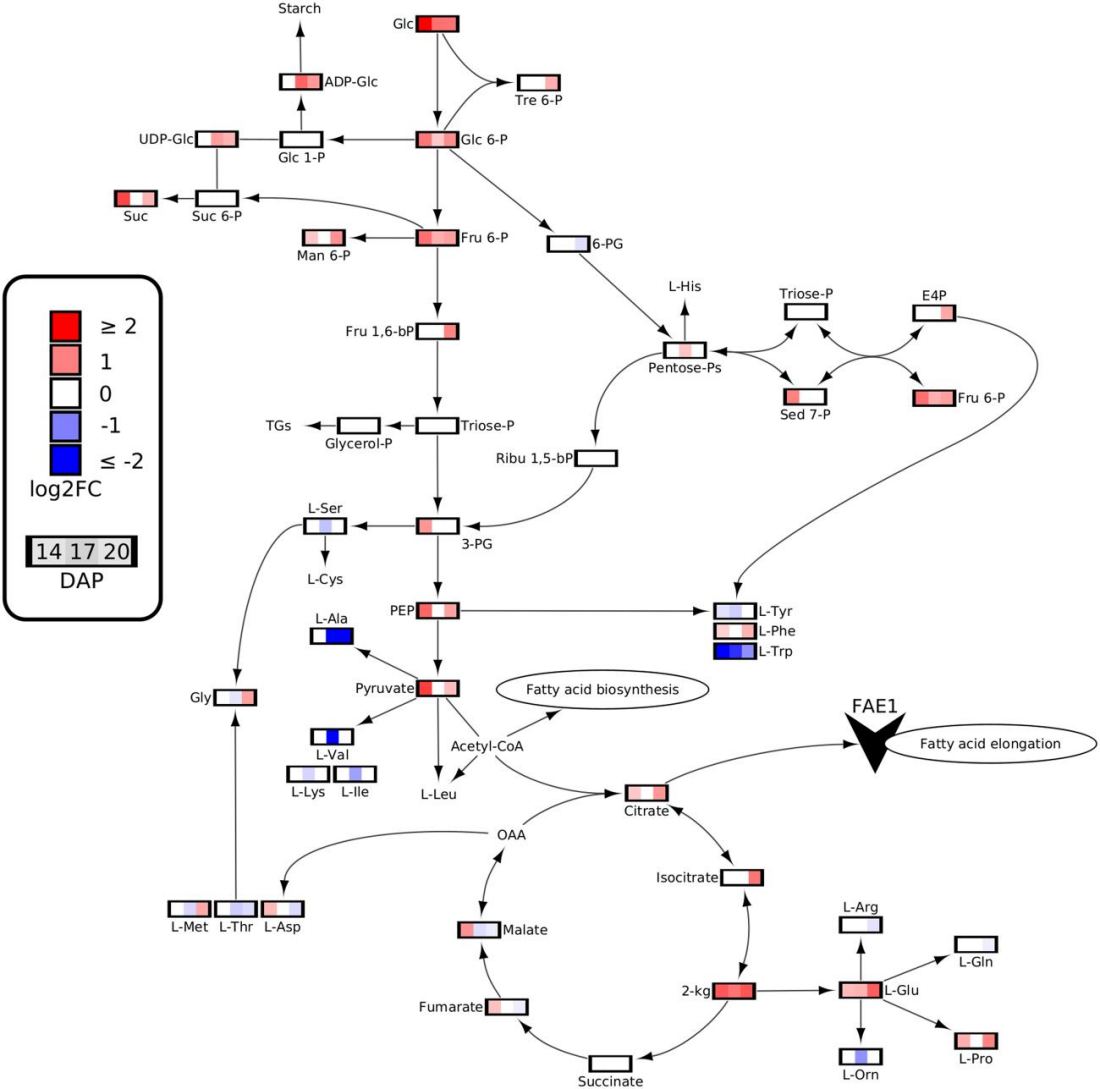
Fold-changes in metabolite quantities throughout central carbon metabolism fold-change of metabolites involved in central carbon metabolism across 3 stages of development 14, 17, and 20 DAP in the WT and *fae1-3* pennycress mutant, *n* = 4. Differential gene expression data were incorporated into Cytoscape; however, it is not presented in the network below as the genes involved were not found to be differentially expressed. Data are in log2FC in *fae1-3* with respect to the WT. –bP, bisphosphate; -P, −phosphate; L-Ala, alanine; L-Arg, arginine; L-Asp, aspartate; E, erythrose; *FAE1*, *FATTY ACID ELONGTION*1; Fru, fructose; Glc, glucose; L-Gln, glutamine; L-Glu, glutamate; Gly, glycine; H, hexose; kg, ketoglutarate; L-Ile, isoleucine; L-Lys, lysine; Man, mannose; L-Met, methionine; OAA, oxaloacetate; L-Orn, ornithine; PEP, phosphoenolpyruvate; PGs, phosphoglycerates; L-Phe, phenylalanine; L-Pro, proline; Ribu, ribulose; Sed, sedoheptulose; L-Ser, serine; Suc, sucrose; TGs, triacylglycerols; L-Thr, threonine; Tre, trehalose; L-Trp, tryptophan; L-Tyr, tyrosine; L-Val, valine.

To gain further insight and contextualize the metabolomics data set, cytoscape was employed to visualize the metabolic pathways within central carbon metabolism (Shannon et al. 2003). Data provided from targeted metabolomics analysis revealed that many intermediaries within central carbon metabolism were augmented by the *fae1* knockout mutation (Fig. 4; Supplementary Table S4). Notably, the content of starch and sucrose precursors such as glucose, glucose 6-phosphate, and fructose 6-phosphate were found to be ∼2 times higher across all developmental stages in *fae1-3*. This coincided with observed elevations in starch and sucrose levels. Additionally, the concentration of the main carbon sources for plastidic and cytosolic acetyl-CoA, namely pyruvate and citrate, were on average 1.7 times higher in developing *fae1-3* embryos.

### Early phase gene expression profiles display shifts in key biological processes such as acyl-CoA metabolism

**Figure 5.**
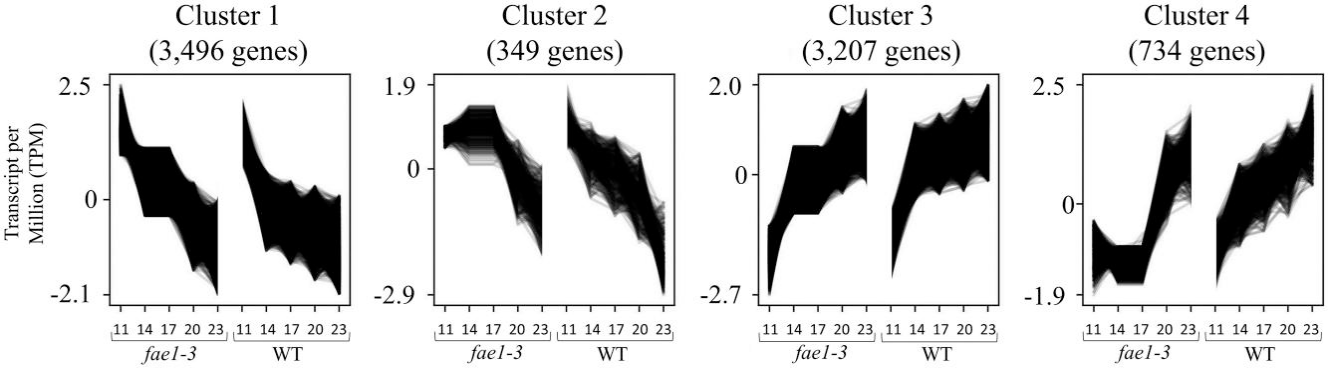
Gene cluster analysis genes were clustered based on expression profiles across 5 developmental time points 11, 14, 17, 20, and 23 DAP using Clust with default parameters, *n* = 3. The *x*-axis displays each time point for the WT and *fae1-3*, while the *y*-axis represents the normalized transcripts per million. Clust automatically determined that the optimal number of clusters for the provided data set is 4, revealing distinct expression patterns across the developmental time course.

In parallel, developing WT and *fae1-3* embryos were collected at 11, 14, 17, 20, and 23 DAP, and RNA was extracted and sequenced as previously described (Johnston et al. 2022; Arias et al. 2023). Clustering analysis of gene expression data were conducted to unveil how genes work in concert to adapt to the loss of VLCFA synthesis. Four distinct gene expression profiles were revealed in this analysis and denoted as Clusters 1 through 4, containing 3,496, 349, 3,207, and 734 genes, respectively (Fig. 5; Supplementary Table S5). The expression profiles of Clusters 1 and 3 did not display obvious qualitative disparities over time between the WT and *fae1-3*; whereas, Clusters 2 and 4 showed distinctive differences. Gene ontology analysis of Clusters 2 and 4 was utilized to gain insight into the functional significance of the genes whose expression profiles were different.

Gene ontology analysis of Cluster 2 revealed an extended early-phase gene expression pattern in *fae1-3* that correspond to specific biological processes, namely acyl-CoA metabolism (GO:0006637, *P-*value = 0.0159), photosynthesis (GO:0015979, *P*-value = 0.0245), and histidine biosynthesis (GO:0000105, *P*-value = 0.0045) (Supplementary Table S6). In Cluster 4, genes associated with intracellular membrane dynamics, specifically vacuole organization (GO:0007033, *P*-value = 2.6E−5), and phospholipid translocation (GO:0045332, *P*-value = 0.0005), had a distinct expression profile between the WT and *fae1-3.* Furthermore, genes involved with defense response (GO:0042742, *P*-value = 0.0012) and protein modification (GO:0036211, *P*-value = 9.7E−8) were also identified as key biological processes that exhibited delayed gene expression in *fae1-3* in Cluster 4.

### Upregulation of genes related to stress response and ATP production is associated with the elimination of VLCFA synthesis

**Figure 6.**
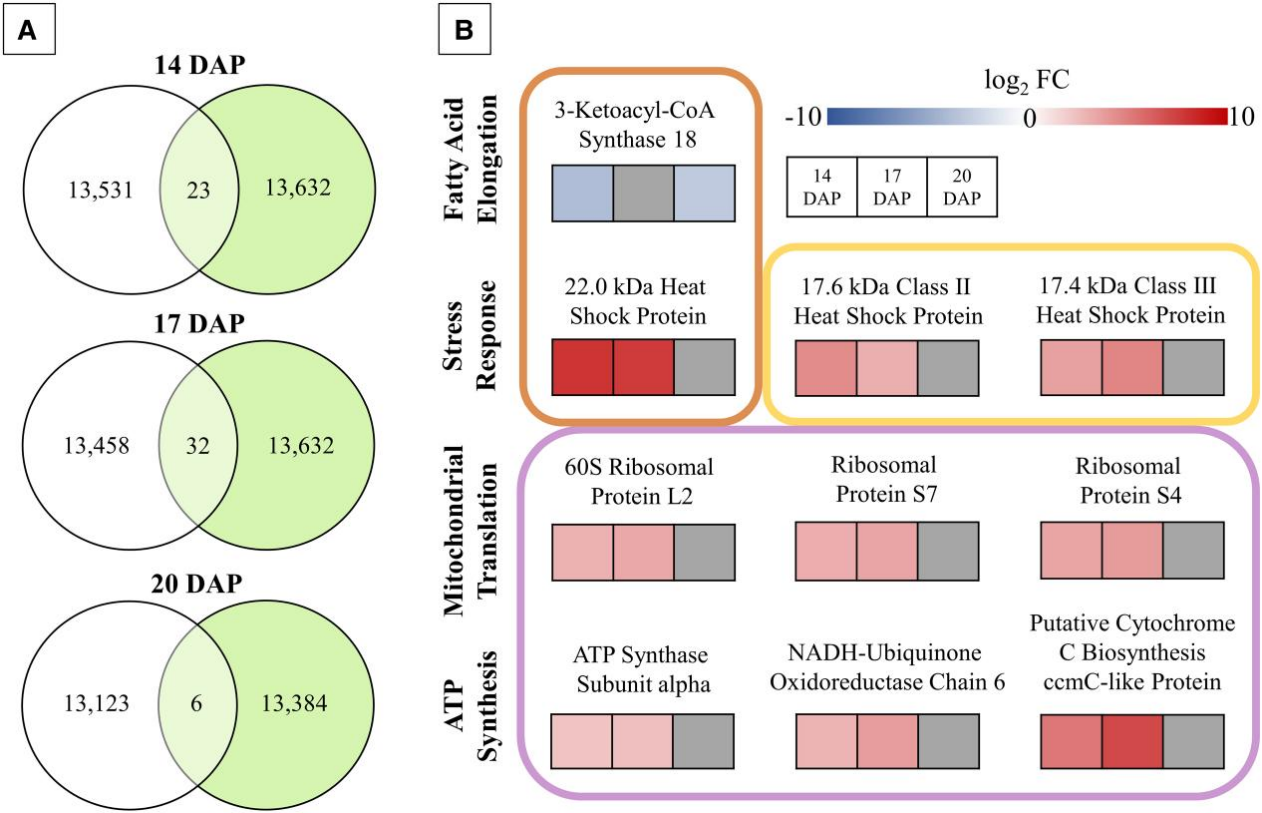
DEGs in developing embryos **A)**. Gene expression profiles of the WT (left circle) and *fae1-3* (right circle) 14, 17, and 20 DAP developing embryos. Genes with a total count above 10 as determined by Salmon were included. Overlap (light green) indicates mutually DEGs as determined by DeSeq2 to be differentially expressed (log_2_FC ≥ 1; *P*-value ≤0.05), *n* = 3. **B)** Genes determined to be differentially expressed across multiple timepoints in developing embryos. For each gene a heat map is utilized to display the log_2_FC in 14, 17, and 20 DAP embryos. As annotated in UniProt, subcellular location is indicated by boxes surrounding gene groups and biological function is delineated by row. Top left box: ER; top right box: cytoplasm; bottom box: mitochondria.

Further analysis of differentially expressed genes (DEGs) were conducted on 14, 17, and 20 DAP WT and *fae1-3* embryos to gain comprehensive insights into the dynamic molecular mechanisms that govern carbon partitioning and metabolic changes during these critical developmental stages. Interestingly, except for *FAE1*, genes encoding for the specific enzymes catalyzing the metabolic reactions shown in Fig. 4 were not identified as being differentially expressed, and thus, not displayed. Despite this, transcriptomics data did reveal some intriguing gene expression patterns associated with the loss of VLCFA synthesis. At 14, 17, and 20 DAP, a total of 23, 32, and 6 genes, respectively, were identified as differentially expressed out of the average total gene count of 13,460 (Fig. 6A). To delve deeper into the relevance of these DEGs, those that appeared at 2 or more developmental stages were functionally characterized and organized by biological function and subcellular localization. As anticipated, the *fae1-3* mutant showed a significant downregulation of *FAE1*, which encodes KCS18 (Fig. 6B; Supplementary Table S7). Additionally, this modification was accompanied by precise upregulation of genes encoding for heat shock proteins (HSPs) associated with cellular stress response, ribosomal proteins related to mitochondrial translation, and several tightly linked with ATP synthesis.

### Joint analysis underscores major impact on cellular respiration and amino acid metabolism

**Figure 7.**
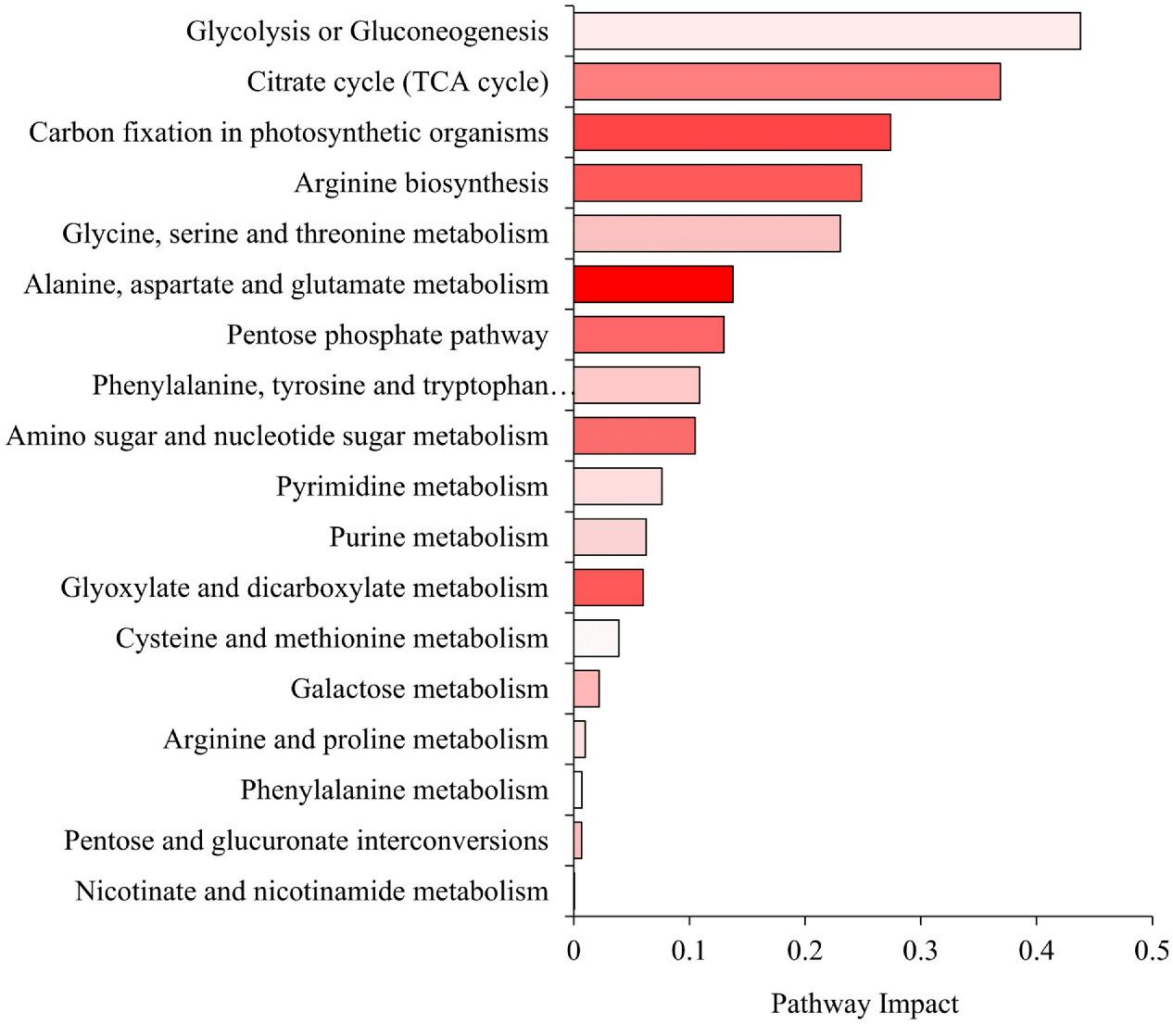
Joint pathway analysis joint pathway analysis to detect pathway enrichment in 14 to 20 DAP developing embryos as determined by MetaboAnalyst using the metabolomics and transcriptomics datasets and pathways from the KEGG database. Pathways shown have a *P*-value ≤0.05 and those above the impact value ≥0.2 were considered significant, *n* = 4 for metabolomics dataset and *n* = 3 for transcriptomics dataset. Shading in a given data bar indicates the −log_10_ (FDR).

Joint pathway analysis, a tool utilized to integrate metabolomics and transcriptomics data sets (Ewald et al. 2024), was employed to identify significantly enriched and impacted pathways in *fae1-3* (*P*-value ≤0.05, impact factor ≥0.2, −log10 (FDR) ≥1.3). The analysis indicated a pronounced impact on central carbon metabolism including key processes such as glycolysis, the TCA cycle, and carbon fixation. It also highlighted pathways integral to amino acid metabolism such as arginine biosynthesis, and glycine, serine, and threonine metabolism (Fig. 7). Among all the pathways, glycolysis emerged as the most affected according to the criteria used (Impact =0.4378, *P*-value = 0.0029, −log_10_ (FDR) = 1.9) (Supplementary Table S8). This finding is characteristic of a concerted cellular effort to cope with and adapt to the alterations caused by/associated with the elimination of VLCFA synthesis.

## Discussion

### Changes in lipid macromolecule composition increases cold-germination efficiency in *fae1* mutants

This investigation revealed a correlation between FA composition and germination rates at temperatures within a range that germinating pennycress seeds experience in fields. In plants, membranes are mainly comprised of glycerolipids containing 2 FA chains at the *sn*-1 and *sn*-2 positions. FA chain length and the degree of saturation influence the fluidity of the plasma membrane and the phospholipid composition is often modulated when encountering abiotic stressors such as temperature changes (Menard et al. 2017; Mironov et al. 2018; Zhukov and Shumskaya 2020). Alterations in FA composition, observed exclusively in the embryos of *fae1* mutant alleles, likely indicate a shift in the molecular species constituting lipid macromolecules. This claim is substantiated by a recent study in pennycress that utilized ESI-MS/MS to quantify the molecular species of phosphatidylcholine in *fae1-3* (Jarvis et al. 2021). A dramatic shift in phosphatidylcholine molecular species profiles were observed demonstrating that a mutation altering FA synthesis and overall FA composition additionally influences the makeup of lipid macromolecules. This lends an explanation to why *fae1-3* and *fae1-4*, containing increased quantities of shorter monounsaturated and polyunsaturated FAs, display increased membrane fluidity at cold temperatures during the water imbibition phase of seed germination (Fig. 1A). Conversely, delayed germination observed at higher temperatures in these mutant alleles could be the result of compromised plasma membrane functionality due to the absence of VLCFAs. It is worth noting that this mutation would not only impact structural components of the plasma membrane, but would also likely influence the structure and therefore function of membrane-bound organelles (Zhukov and Shumskaya 2020). This effect on the cellular membranes is evidenced by the distinct expression profiles of genes associated with phospholipid translocation and vacuole organization observed in ontology results (Fig. 5; Supplementary Table S6). Overall, VLCFAs appear to be beneficial for high-temperature tolerance, as evidenced by the faster germination of the WT at elevated temperatures. This suggests that VLCFAs play a critical role in withstanding heat stress. The mutant seeds, which lack VLCFAs exhibited faster germination under cold conditions. This observation implies that the long chain monounsaturated and polyunsaturated FAs, which are indeed more abundant in the *fae1* mutant, likely, contribute to cold-tolerance.

### Overexpression of key acyltransferases could balance reduced triacylglycerol content in composition mutants

As aforementioned, there was a significant shift in the FA composition of *fae1* embryos (Figs. 1A and 2B). This shift did not impact the content of total FAs, as shown in this investigation; however, another study conducted in *fae1* embryos revealed reduced triacylglycerol (TG) content in mature *fae1* embryos, suggesting a particular issue with lipid storage (Jarvis et al. 2021). Reduced TG content observed in lipid composition mutants could be attributed to increased β-oxidation and glyoxylate cycle activity. However, transcripts encoding key enzymes directly involved in β-oxidation, including acyl-CoA oxidases, enoyl-CoA hydratases, and 3-ketoacyl-CoA thiolases, as well as those associated with the glyoxylate cycle, such as malate synthase and isocitrate lyase, were not upregulated in the *fae1-3* mutant. Therefore, it is much more likely that the decrease in TG content is the result of feedback inhibition of diacylglycerol acyltransferase (DGAT) triggered by the increase of oleic acid and other long chain FAs. Activity assays conducted on *Brassica napus DGAT1* illustrated the role of the N-terminal domain as a negative modulator of TG biosynthesis (Caldo et al. 2017). Furthermore, in mammalian systems long chain acyl-CoAs have been shown to play a regulatory role in lipid metabolism (Hu et al. 2005; Nagy et al. 2014). Looking forward, to ensure TG content remains uncompromised, it may be prudent to enhance the expression of key acyltransferases involved in TG biosynthesis, such as DGAT, alongside strategic modifications aimed at enhancing FA composition (Parchuri et al. 2022; Sagun et al. 2023).

### Reduced storage lipid content impacts cellular energy balance triggering metabolic shifts and starch accumulation

TGs, containing twice the energy content of storage carbohydrates, play an essential role as carbon and energy reserves in developing plant embryos (Zhang et al. 2010; Ohlrogge and Chapman 2011). Accordingly, FA biosynthesis and TG assembly are energetically expensive costing the cell high quantities of ATP and reducing equivalents (Baud and Lepiniec 2010; Sagun et al. 2023). This aligns with the correlation between reduced TG content and the observed increase in ATP quantity in *fae1-3*. Additionally, the energy charge determined by the ratio of ATP, ADP, and AMP is elevated in *fae1-3*; calculated to be 0.49 for the WT and 0.56 for *fae1-3* (Supplementary Table S4). This indicates reduced consumption and/or increased energy production (Atkinson and Walton 1967; De La Fuente et al. 2014). Consistent with this, genes related to energy production were upregulated in *fae1-3* (Fig. 6B; Supplementary Table S7) including genes encoding proteins involved in mitochondrial translation; a critical process for synthesizing proteins involved in oxidative phosphorylation (Aibara et al. 2020). Furthermore, genes encoding proteins directly associated with ATP biosynthesis such as ATP synthase subunit alpha were also upregulated in *fae1-3*. Taken together, these findings suggest that modifications to FA composition lead to disturbances in cellular energy balance. This is further substantiated by the understanding that FAE is an ATP-dependent process as elucidated in (Heil et al. 2019).

These disruptions to cellular energy homeostasis subsequently trigger dramatic shifts in central carbon metabolism as evidenced by the increase of glucose and ADP-glucose quantities (Fig. 4; Supplementary Table S4). The surplus availability of sugar precursors likely contributes to the increased accumulation of starch in *fae1-3* (Fig. 2A). Starch, a complex polysaccharide, serves as a crucial reservoir of carbon and energy in seeds (Streb and Zeeman 2012). By directing excess carbon toward starch synthesis, *fae1-3* exhibits its ability to respond to altered energy requirements ensuring adequate reserves are readily available to the developing embryo for future germination. This metabolic shift underscores the embryo’s adaptive capacity to dynamically respond to physiological changes. Moreover, in mutants with altered FA composition, redirecting carbon flow away from other biomass components such as starch could be vital in preventing a decrease in oil content (Tsogtbaatar et al. 2020).

To reduce starch accumulation and redirect more carbon toward oil production during development, several strategies could be implemented. One approach to reducing excess starch during embryo development is to enhance starch degradation by upregulating genes like α-amylase or β-amylase. This strategy could be effective, as high α-amylase activity has been linked to low starch content in wheat embryos (Umemura et al. 1998). Another strategy is to boost FA biosynthesis by overexpressing *WRINKLED1* (*WRI1*), a key regulator of oil biosynthesis, shifting carbon allocation from starch to lipid production (Grimberg et al. 2020). However, the most optimal approach may involve targeting both starch and FA biosynthesis by combining reduced AGPase expression with upregulated *WRI1*. This dual modification has been shown to reduce starch accumulation, leading to up to a 5.8-fold increase in oil production, compared with plants with alterations in either gene alone (Sanjaya et al. 2011). This strategy could offer a balanced modification, optimizing carbon allocation in embryos by enhancing oil accumulation while minimizing unwanted starch buildup. This comprehensive analysis identified complex changes in lipid metabolism, homeostasis, and stress responses providing insight for future improvement of pennycress oil composition while maintaining oil yield and seed health.

### Alterations in lipid biosynthesis elicits a cellular stress response

The upregulation of genes encoding HSPs, often referred to as stress response proteins, was observed in *fae1-3* (Fig. 6B; Supplementary Table S7). These proteins play vital roles in maintaining proteostasis in plants under stress (Shan et al. 2020; Wu et al. 2022). In adverse conditions, HSPs act as chaperones, involved in stabilizing and refolding denatured proteins, preventing protein aggregation, and facilitating the degradation of damaged proteins (Waters and Vierling 2020). The heightened expression of HSP genes in *fae1-3* implies the activation of a compensatory mechanism aimed at alleviating cellular stress initiated by alterations in lipid composition (Lamers et al. 2020; Zhao et al. 2020). Likely, as various plasma membrane-localized lipids have been identified as stress sensors, the absence of VLCFAs triggers a stress signaling response (Lamers et al. 2020). Further elucidation of this stress signaling pathways holds promise to improve our understanding of plant stress perception and the role of lipids in stress response signaling. Additionally, it could lead to the discovery of genetic targets aimed at bolstering plant resilience to various abiotic stressors like heat and drought.

## Materials and methods

### Chemicals

For plant husbandry, Murashige and Skoog (MS) salt medium, Gamborg’s vitamin solution, and Gibberellins (GA_4_/GA_7_) were sourced from PhytoTechnology Laboratories (Lenexa, KS). For the extraction, methylation, and quantification of FAs the standard glyceryl triheptadecanoate and chemicals toluene, methanolic HCl, and sodium hydrogen sulfate (NaHSO_4_) were obtained from Sigma-Aldrich (St. Louis, MO), while hexane, 2-propanol, and methanol were sourced from Thermo Fisher Scientific (Waltham, MA). Protein extraction and quantification were conducted using a solution containing Tris(hydroxymethyl)aminomethane, sodium chloride (NaCl), and sodium dodecyl sulfate (SDS) from Thermo Fisher Scientific (Waltham, MA), in conjunction with the DC Protein Assay Reagents from Bio-Rad (Hercules, CA). Starch extraction and quantification procedures involved the use of glacial acetic acid from Sigma-Aldrich (St. Louis, MO) and amyloglucosidase and D-glucose standard from Megazyme (Bray, IE). Regarding quantification of metabolites, external metabolite standards were ordered from MilliporeSigma while solvents for GC-MS and LC-MS/MS were procured from Thermo Fisher Scientific (Waltham, MA). Internal standards including [U-^13^C_2_]-glycine, [U-^13^C_6_]-glucose, and [U-^13^C_4_]-fumarate were sourced from Cambridge Isotope Laboratories (Tewksbury, MA). RNA extraction was carried out using RNase-free water and glycogen from Thermo Fisher Scientific (Waltham, MA), hexadecyltrimethylammonium bromide (CTAB), 2-mercaptoethanol, polyvinylpolypyrrolidone, chloroform, ethylenediaminetetraacetic acid, sodium acetate, and lithium chloride from Sigma-Aldrich (St. Louis, MO), spermidine from Alfa Aesar (Ward Hill, MA), isoamyl-alcohol from EM Science (Hatfield, PN), and ethanol from Decon Labs Inc. (King of Prussia, PA).

### Plant materials and husbandry

Pennycress (*T. arvense*) seeds from the WT Spring 32-10 accession originating from Bozeman, Montana (Lat: 45.664, Long: −111.048, Alt: 1496 m) were kindly provided by the Arabidopsis Biological Resource Center (Columbus, OH). Spring 32-10 was utilized to generate the *fae1-3* and *fae1-4* mutant alleles by an Agrobacterium-mediated floral dip transformation method and CRISPR-Cas9 technology as previously described (McGinn et al. 2019). The *fae1-3* and *fae1-4* mutant alleles contain a 4 base-pair deletion and 1 base-pair insertion in the *FAE1* coding sequence, respectively, constituting knockout alleles based on the absence of erucic acid production. Seeds were first germinated in petri dishes under sterile conditions before potting (Tsogtbaatar et al. 2015). The seeds were sterilized for 5 min. with 30 mL of 50% bleach in a 50 mL falcon tube and then rinsed with sterile water a total of 4 times. Following seed sterilization, the seeds were placed on aseptic Whatman papers in a 100 × 15 mm glass Petri dish. The Whatman papers were immersed in 3 mL of germination media containing filter-sterilized MS salt medium and 1 mm G4/G7 gibberellins (pH 6.3 to 6.5). Approximately 30 seeds were plated and the petri dish was sealed with parafilm. Seeds were allowed to germinate for 5 d at 22 °C. Germinated *T. arvense* seeds were then transferred into 13 cm diameter and 13 cm height pots. Two plants were allowed to grow in each pot. Following potting, plants were grown in a greenhouse at 21 to 22 °C with a light intensity of ∼300 µmoles m^−2^ s^−1^ and a 16 h/8 h day/night cycle. Both the WT Spring 32-10 pennycress accession and mutant *fae1-3* and *fae1-4* were kept continuously under typical growth conditions as vernalization is not required to induce flowering for this accession of pennycress. In order to prevent cross pollination, a pollen screen was utilized between WT and mutant plants. Five branches were continuously maintained for experiments; all other branches were pruned. Each day, plants were watered and emerging flowers were pollinated and tagged to keep account of the developmental stage of the embryos (Tsogtbaatar et al. 2015). Biological replicates (*n* = *x*) refer to samples collected from different plants within the same experiment, ensuring the data captured biological variation.

### Germination and physiology measurements

#### Seed germination at various temperatures

Germination rate and efficiency of pennycress seeds was investigated across a range of temperatures, namely 10, 16, 22, 28, and 34 °C. Each treatment involved placing 3 replicates of 25 seeds on Petri dishes lined with filter paper and moistened with 3 mL of germination media, as described in “Plant Materials and Husbandry.” To ensure consistency, the Petri dishes were covered in foil to eliminate the impact of light variability and placed in growth chambers under controlled temperature settings. The emergence of the radicle reaching 2 mm from the seed coat was defined as complete seed germination, which was recorded daily for a period of 12 d (protocol adopted from Maraghni et al. 2010).

#### Plant morphology

This experiment involved measuring various growth and reproductive characteristics of pennycress plants. The seeds were germinated and plants were grown according to the “Plant Materials and Husbandry” section, with some modifications, such as growing only 1 plant per pot and refraining from pruning branches or tagging flowers. The experiment utilized 10 replicates for each line (the WT and 2 *fae1* alleles). The measured variables during development included the number of leaves and plant height once a week until maturity using protocols adopted from (Dalal et al. 2015).

#### Photosynthetic measurements

Gas exchange measurements of leaves and silicles were monitored using a LI-6800 Portable Photosynthesis System (LI-COR Biosciences, Lincoln, NE) under greenhouse conditions as outlined in the “Plant Materials and Husbandry” section. The experiment involved 4 biological replicates for each line (WT and 2 *fae1* alleles). The leaf measurements were collected from the mid-portion of the leaf blade; while the silicle measurements were taken from 17 DAP pods.

### Embryo collection

Pennycress embryos were collected at 5 distinct developmental stages: 11, 14, 17, 20, and 23 DAP, and at a consistent time, between 12:00 and 2:30 PM, to avoid differences caused by diurnal cycle. For each of these developmental stages, we harvested 4 biological replicates for targeted metabolomics and 3 replicates for transcriptomics analyses. The number of embryos per replicate at these respective stages were 50, 20, 15, 10, and 6. The developmental time points 14, 17, and 20 DAP were deliberately selected for the majority of data analyses and interpretations as this timeframe corresponds to linear biomass accumulation (Tsogtbaatar et al. 2015; Johnston et al. 2022). The silicles were harvested and kept on ice then incised under a dissecting microscope so that the embryo could be removed from all maternal plant tissue. For biomass and metabolomics analyses, the embryos were collected into 2 mL microcentrifuge tubes on ice and then lyophilized at −80 °C for 3 d to obtain the DW. For transcriptomics analyses, all materials used for embryo collection were autoclaved and treated with RNase Zap (MilliporeSigma, Burlington, MA). Collected embryos were immediately transferred into liquid nitrogen then stored at −80 °C until further analysis.

### Biomass quantification

FAs were extracted and methylated from freeze-dried pennycress embryos as described in (Cocuron et al. 2014). The quantification of FA methyl esters was conducted with an Agilent 6890N gas chromatograph (Agilent Technologies, Santa Clara, CA) coupled to an Agilent 5975B mass selective detector in accordance with (Tsogtbaatar et al. 2015). The column, specific instrument parameters, and software utilized to acquire integration data are described in (Johnston et al. 2022). Protein was extracted from the remaining pellet using a protein extraction buffer (20 mm Tris-HCl, 150 mm NaCl, and 1% (w/v) SDS) as outlined in (Cocuron et al. 2014). Extracted proteins were then quantified using a DC protein colorimetric assay (Bio-Rad, Hercules, CA) and Bio-Rad SmartSpec Plus spectrophotometer as described in (Johnston et al. 2022). Starch was then extracted from the remaining pellet in accordance with (Cocuron et al. 2014), and glucosyl units cleaved from starch were quantified by LC-MS/MS as described below.

### Water-soluble metabolite extraction

A boiling water extraction was conducted on developing pennycress embryos to extract relevant water-soluble metabolites including amino acids, sugars and sugar alcohols, organic acids, and phosphorylated compounds. The freeze-dried embryos were initially homogenized in a mixer mill (Retsch M400, RETSCH, Haan, Germany) using 5 mm metal beads at 30 Hz for 2 min. Homogenized samples were then immediately transferred on ice to a refrigerated centrifuge (XTR1, Thermo Fisher, Walthum, MA) set at 4 °C and centrifuged at 17,000 × *g* for 30 s. Following centrifugation, a standard solution including 10 µL of 4 mm [U-^13^C_2_]-glycine, 10 mm [U-^13^C_6_]-glucose, 1 mm [U-^13^C_4_]-fumarate was added to each sample and in a separate control microcentrifuge tube to account for any losses during sample extraction and preparation. As described in (Alonso et al. 2010; Cocuron et al. 2014), water-soluble metabolites were extracted with boiling deionized water. Lastly, extracts were filtered and freeze-dried overnight then resuspended in 1 mL of ice-cold water and additionally filtered before LC-MS/MS analysis in accordance with (Johnston et al. 2022).

### LC-MS/MS quantification of water-soluble metabolites

All metabolites were analyzed by a 1,290 Infinity II ultra-high-performance liquid chromatograph (Agilent Technologies, Santa Clara, CA) coupled with a QTRAP 6500+ mass spectrometer (SCIEX, Framingham, MA). Methods for the analysis of amino acids, sugars and sugar alcohols, and organic acids and phosphorylated compounds are detailed elsewhere (Alonso et al. 2010; Koubaa et al. 2013; Cocuron et al. 2014; Cocuron and Alonso 2014). Sample dilutions, column utilization, and ionization modes for each class of compounds are described in (Johnston et al. 2022). LC-MS/MS data were acquired and processed using MultiQuant software (Sciex, Framingham, MA). Metabolomics statistical analyses including the PLS-DA and VIP was conducted using MetaboAnalyst 6.0 (Ewald et al. 2024).

### RNA extraction and purification

RNA was extracted from frozen pennycress embryos in accordance with (Johnston et al. 2022). Following the RNA extraction and resuspension in RNase free-water, DNA contamination was eliminated using the RNase-Free DNase Set (Qiagen, Hilden, Germany). RNA purification following enzymatic treatment was carried out using the QIACube Connect equipped with an RNeasy MinElute Cleanup Kit (Qiagen, Hilden, Germany). To evaluate the quantity and quality of the RNA the QUBIT 3.0 fluorometer (Invitrogen, Carlsbad, CA) and Agilent 2100 Bioanalyzer paired with the Agilent 4200 TapeStation (Agilent Technologies, Santa Clara, CA) were utilized. Samples were shipped on dry ice and RNAseq was performed using DNBSeq technology (BGI, Cambridge, MA).

### RNAseq data preparation and analysis

An average of 33.2 million reads per sample was obtained after confirming the quality with FastQC ([CSL STYLE ERROR: reference with no printed form.]). All paired-end reads were mapped to the *T. arvense* genome (GenBank Acc.: GCA_911865555.2), (Nunn et al. 2022) using HISAT2 v. 2.2.1 (Kim et al. 2019) and sorted with Samtools (Li et al., 2009). Transcripts were assembled using StringTie v. 2.2.1 (Pertea et al. 2016) using GenBank Acc.: GCA_911865555.2 as the reference annotation to guide assembly. The resulting assembly was converted into FASTA format with gffread (Pertea and Pertea 2020). A transcript-to-gene matrix was generated with AGAT (Dainat et al. 2023), with genes defined as locations on the genome from which transcript isoforms are transcribed. Filtering of duplicates was carried out using both the tr2aacds script from EviGene (Gilbert 2023) and CD-HIT-EST (Li et al. 2001) with a cutoff of 0.95. Finally, chimeras were detected and removed using the method described in (Yang and Smith 2013). The filtered transcript assembly contained 30,757 total sequences with a mean length of 1,404 bp. Analysis of the filtered transcript assembly with BUSCO (Manni et al. 2021) indicated that 93.9% of genes in the “brassicales_odb10” database were represented. BLAST (Altschul et al. 1990) was used to assign homologs from the *A. thaliana* database (UPID: UP000006548) from UniProt (The UniProt Consortium 2019). The quasi-mapping mode of Salmon (Patro et al. 2017) was then used to obtain raw counts used for transcript quantitation. Counts were imported into R using Tximport (Soneson et al. 2016) prior to detection of DEGs and differentially expressed transcripts using DeSeq2 (Love et al. 2014) at a cutoff of *P*_adj_ = 0.01 and log_2_FC ≥ |1|. Genes were clustered based on expression profiles across 5 developmental time points (11, 14, 17, 20, and 23 DAP) using Clust with default parameters (Abu-Jamous and Kelly 2018). The Clust function was allowed to automatically determine the optimal number of clusters (Johnston et al. 2024). Gene ontology analysis using g. Profiler (https://biit.cs.ut.ee/gprofiler/gost) of the identified clusters was then carried out (Raudvere et al. 2019).

### Statistical and joint pathway analysis

A *t*-test was performed to compare the means of 2 independent groups to assess the significance of observed differences. For comparisons involving more than 2 groups, a one-way ANOVA was conducted to determine whether significant differences existed among group means. Post hoc analyses were performed using Tukey’s honest significant difference test to identify pairwise differences while controlling for multiple comparisons. Statistical significance was determined at a threshold of *P*-value ≤ 0.05.

MetaboAnalyst 6.0 was used to perform statistical analyses of the metabolomics data set and joint pathway analysis integrating metabolomics and transcriptomics datasets obtained from embryos at the 14 to 20 DAP developmental stage (Ewald et al. 2024; Johnston et al. 2024). Statistical analysis of targeted metabolomics data involved a log transformation and auto scaling which entailed mean-centering and dividing by the Sd of each feature. The approach was utilized to mitigate the influence of metabolites with high abundances, ensuring less abundant metabolites contributed meaningfully to the analysis. Normalization was not applied because absolute quantities were used. Joint pathway analysis incorporated features (metabolites or transcripts) that were significantly different between *fae1-3* and WT embryos. These differences were identified either as DEGs for transcripts or as metabolites with a significance of *P* ≤ 0.05 for any developmental stage between 14 and 20 DAP.

Enrichment analysis was conducted using a hypergeometric test, with betweenness centrality as the topology measure. To integrate the features, *P*-values were combined via loose integration such that metabolite and transcript data sets were equally weighted.

### Accession numbers

The data discussed in this publication have been deposited in NCBI’s Gene Expression Omnibus (Edgar et al. 2002) and are accessible through GEO Series accession number GSE283023 (https://www.ncbi.nlm.nih.gov/geo/query/acc.cgi?acc=GSE283023).

## Supplementary Material

kiae650_Supplementary_Data

## Data Availability

The data underlying this article are available in NCBI's Gene Expression Omnibus and are accessible through GEO Series accession number GSE283023 (https://www.ncbi.nlm.nih.gov/geo/query/acc.cgi?acc=GSE283023).

## Supplementary data

The following materials are available in the online version of this article.

**Supplementary Figure S1.** Biosynthetic pathways involved in the elongation and desaturation of FAs.

**Supplementary Figure S2.** Plant morphology and photosynthetic measurements.

**Supplementary Figure S3.** Acetyl-units in developing and mature embryos.

**Supplementary Table S1.** Mature seed biomass composition.

**Supplementary Table S2.** Germination efficiency comparison at various temperatures.

**Supplementary Table S3.** Developing embryo biomass composition.

**Supplementary Table S4.** Metabolite quantity in developing embryos.

**Supplementary Table S5.** Clust gene identities.

**Supplementary Table S6.** Gene Ontology analysis.

**Supplementary Table S7.** DEGs in developing embryos.

**Supplementary Table S8.** Joint pathway analysis.

## Dive Curated Terms

The following phenotypic, genotypic, and functional terms are of significance to the work described in this paper:

KCS18 Gramene: AT4G34520
KCS18 Araport: AT4G34520

starch CHEBI: CHEBI:28017

NADPH CHEBI: CHEBI:16474

ATP CHEBI: CHEBI:15422

BUSCO Gramene: Benchmarking Universal Single-Copy Orthologs
BUSCO Araport: Benchmarking Universal Single-Copy Orthologs

acetyl-CoA CHEBI: CHEBI:15351
