# Supplementary material for: Propelling sustainable energy: Multi-omics analysis of pennycress FATTY ACID ELONGATION1 knockout for biofuel production
Source: Plant Physiol. 2024 Dec 9;197(2):kiae650. doi: 10.1093/plphys/kiae650 (PMC11809582; doi:10.1093/plphys/kiae650)
Supplement: kiae650_Supplementary_Data [file kiae650_supplementary_data.zip › Supplemental Figures PDF.pdf]

Supplemental figure S1.

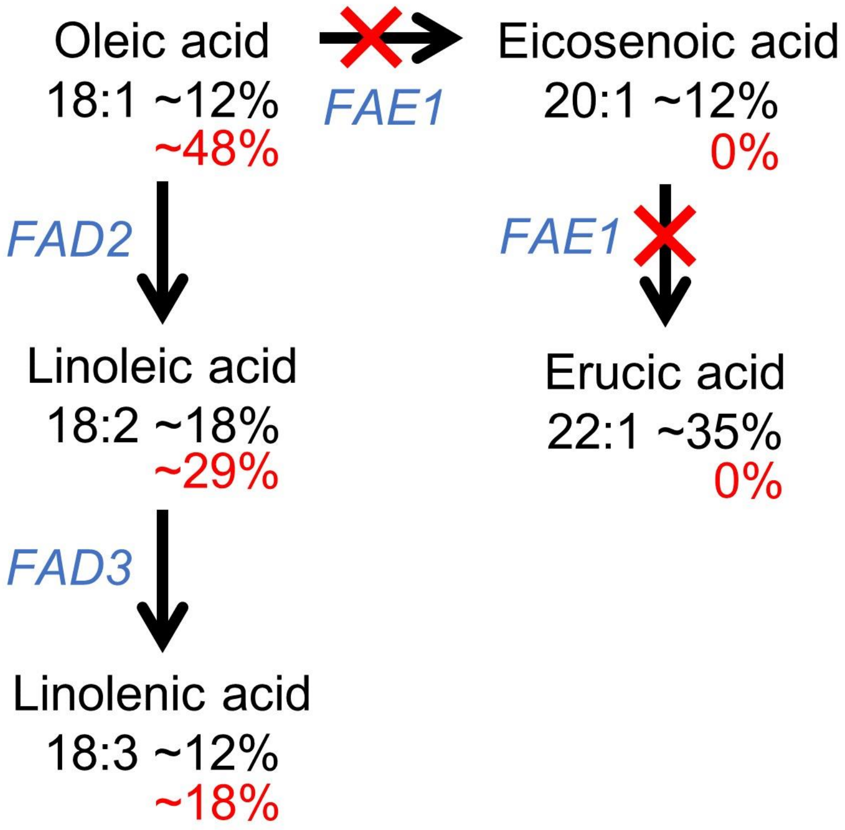

Supplemental figure S1. Biosynthetic pathways involved in the elongation and desaturation of fatty acids | Simplified schematic of biosynthetic pathways involved in the elongation and desaturation of fatty acids that become incorporated into triacylglycerides (TGs). Percentages represent the weight percent proportions of fatty acids in TGs from the seeds of wildtype (WT) (black) versus *fatty acid elongation 1* (*fae1*) knockout mutant (red). Note that *FAE1* is part of a protein complex that sequentially adds two carbons to 18:1-CoA then 20:1-CoA to form 22:1-CoA (erucic acid). *FAD 2,3, FATTY ACID DESATURASE 2,3.*

Supplemental figure S2.

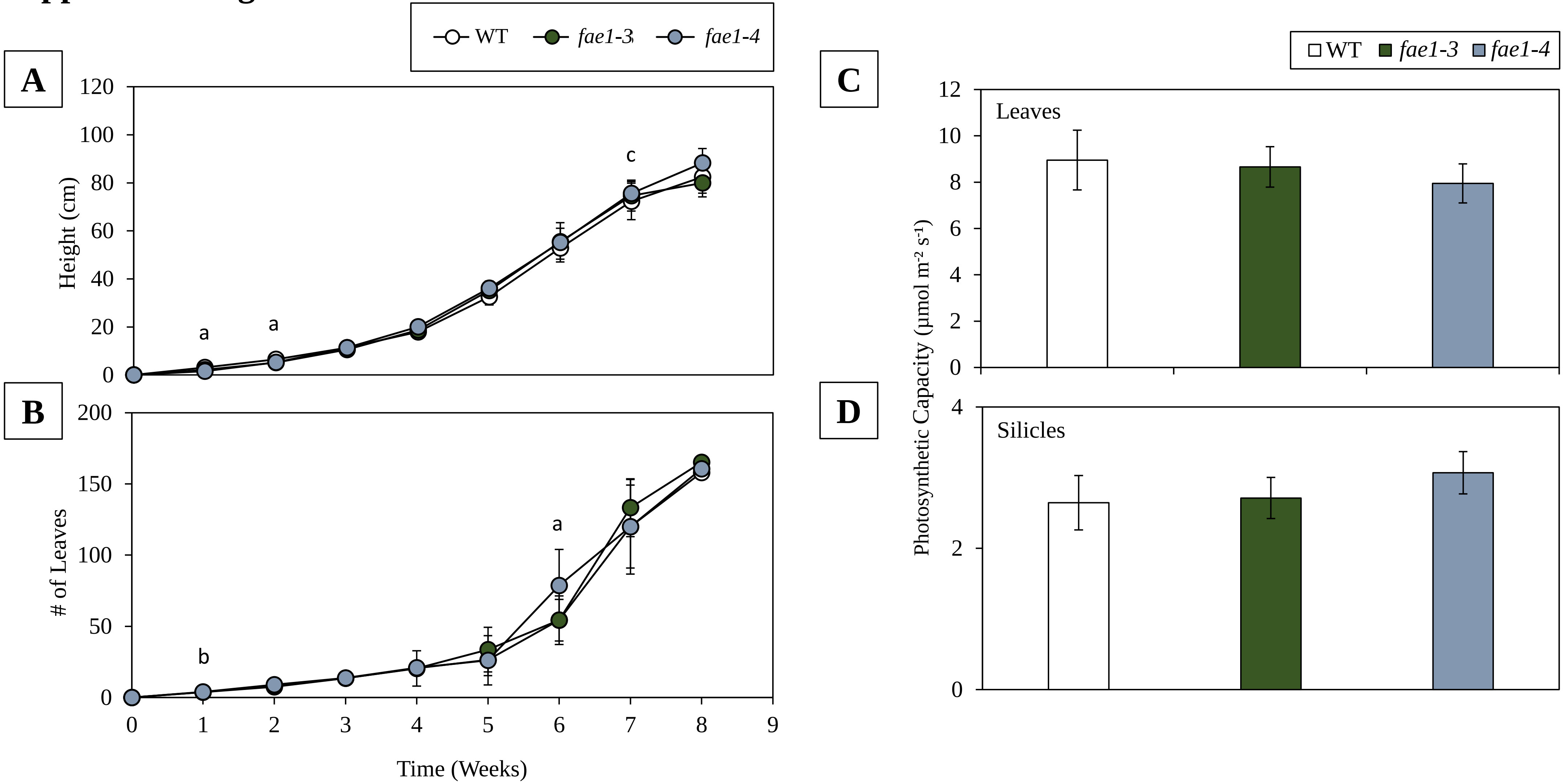

Supplemental figure S2. A. Height and B. leaf number were compared during development between the wildtype (WT) and two *fatty acid elongation 1* (*fae1*) mutant alleles (*fae1-3* and *fae1-4*). Significant differences are marked as follows: "a" indicates differences between the WT and both *fae1* alleles, "b" indicates differences between the WT and a single *fae1* allele, and "c" indicates differences between the *fae1* alleles. C. Photosynthetic measurements were taken for leaves and D. 17 days after pollination (DAP) silicles in the WT, *fae1-3*, and *fae1-4*. Group differences are denoted by letters. Statistical significance ($p \leq 0.05$) was assessed using ANOVA and Tukey's HSD test. Error bars represent standard deviations of biological replicates: A, B (n=10); C, D (n=4).

Supplemental figure S3.

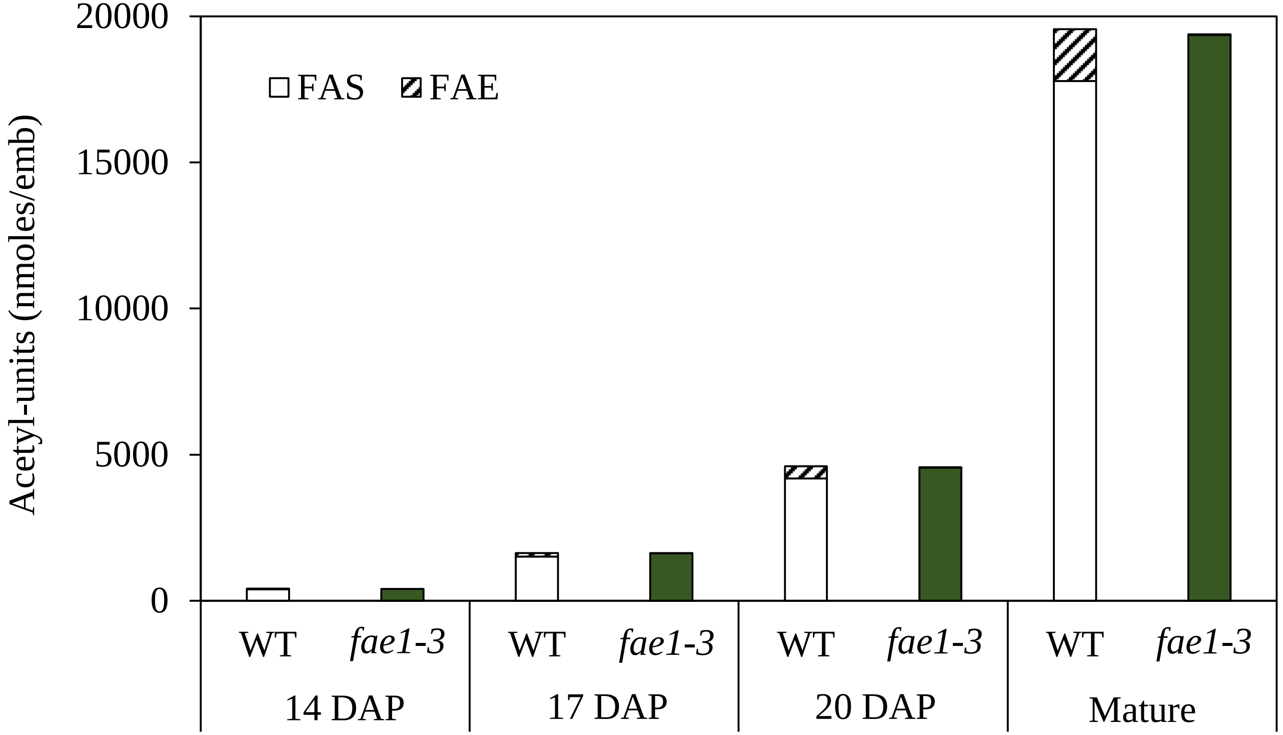

Supplemental figure S3. Acetyl-units in developing and mature embryos **|** Acetyl-units in nmol/embryo for the wildtype (WT) (white) and *fatty acid elongation 1-3* (*fae1-3*) (green) mutant embryos at three developmental stages: 14, 17, and 20 days after pollination (DAP), and at maturity. The bars without stripes represent the amount of acetyl-units entering fatty acid synthesis, while the bars with diagonal lines show the acetyl-units allocated to elongation. A t-test was applied to assess the differences between groups at each stage. The absence of asterisks indicates no significant difference at ($p \leq 0.05$), n=4 (14-20 DAP), 10 (Mature).
