# Supplementary material for: Propelling sustainable energy: Multi-omics analysis of pennycress FATTY ACID ELONGATION1 knockout for biofuel production
Source: Plant Physiol. 2024 Dec 9;197(2):kiae650. doi: 10.1093/plphys/kiae650 (PMC11809582; doi:10.1093/plphys/kiae650)
Supplement: kiae650_Supplementary_Data [file pp.02686.2024-s07.pdf › pp.02686.2024-s07.pdf]

Plant Physiology®

Propelling sustainable energy: Multi-omics analysis of pennycress fatty acid elongase 1 knockout for biofuel production

Amira Rasoul, Christopher Johnson, Jordan LaChance, John Sedbrook, and Ana Paula Alonso

Review Timeline:

Submission Date:	03-Jun-2024
Editorial Decision:	07-Aug-2024
Resubmission Received:	17-Oct-2024
Accepted:	15-Nov-2024

02-Aug-2024

Dr. Ana Paula Alonso
University of North Texas
BioDiscovery Institute
Denton, Texas 76201

RE: Propelling sustainable energy: Multi-omics analysis of pennycress fatty acid elongase 1 knockout for biofuel production

Dear Dr. Ana Paula Alonso:

Thank you for giving us the opportunity to review your manuscript for publication in the Plant Physiology. The reviewers have found the manuscript of interest, but have raised a number of concerns that will require your attention. As these concerns are of some gravity and will entail substantial changes to the manuscript, you are likely to need additional time to address them. I must therefore reject the manuscript in its present form. Nonetheless, if you are able to address the points raised in review, I would welcome your resubmission of a manuscript that incorporates your responses.

In particular, I direct your attention to the following comments for consideration in a resubmitted manuscript.

Better justify the novelty of the study.

Explain the quantitative differences in fatty acids between WT and mutant

I agree with the reviewer, unusual fatty acid has different behavior for the acyltransferase substrate. Try to bring other experiemental evidences.

Also answer all the other points raised by the 2 referees

Please note that any resubmission will be treated as a new entry to the system and is likely to go out for review.

If you do have any queries or concerns, you are welcome to contact me in the first instance.

Thank you again for allowing Plant Physiology to review your work. We look forward to hearing from you soon.

Sincerely,

Editor, Plant Physiology

-- Reviewer comments:
Reviewer #1 (Comments for the Author):

The paper entitled "Propelling sustainable energy: Multi-1 omics analysis of pennycress fatty acid elongase 1 knockout for biofuel production" describes the metabolic and physiological characterization of the fae1 mutant in Thlaspi arvense L. (field pennycress), a non-model plant with potential applications in the biofuel sector. This manuscript combines transcriptomics and metabolomics data from developing and mature seeds, focusing on fatty acid profiles. While the study is well-written, certain aspects require clarification before publication.

Given previously published papers on fae1 mutants (Jarvis et al., 2021; Batsale et al., 2021; Ozsayhan et al., 2017), the authors should emphasize and justify the novelty of their study.

The research reveals that from 14 to 20 days after pollination, no significant quantitative differences in fatty acids were observed between WT and mutant developing embryos. However, in mature ones, both quantitative and qualitative differences were identified. Could you please explain these differences?

The authors suggest that the reduction in triglycerides in mature embryos (not observed in developing embryos) results from acyltransferases' inability to handle the accumulation of medium-chain fatty acids. They support this conclusion by referencing Bates et al. (2014), which demonstrated inefficient incorporation of hydroxyl fatty acids. However, comparing these metabolic situations may be problematic, as Bates' study involved an unusual fatty acid as the acyltransferase substrate. As they mentioned, overexpression of key acyltransferase could clarify this point.

Reviewer #2 (Comments for the Author):

Dear Authors,

This study provides detailed insights into the effect of FAE1 mutants on fatty acid (FA) composition and their impact on improving the cold-flow properties of the oil for sustainable aviation. The use of multi-omic approaches, including metabolomic and transcriptomic datasets on developing embryos, is particularly interesting. Additionally, the observation that fae mutants upregulated heat shock proteins is noteworthy. Experiments exploring the role of very long-chain fatty acids (VLCFAs) in heat tolerance using fae mutants would strengthen the paper, as would further investigation into cold tolerance, given that the mutants germinated well and earlier compared to wild types.

I have provided detailed comments in the attached version. The discussion section needs to be re-written. Much of it reiterates the introduction and results; instead, it should focus on the impact of the study and future directions. Therefore, I recommend a major revision.

Modifications and responses to points raised by the reviewers

We would like to thank the reviewers for their careful work and useful corrections and suggestions. To facilitate the review process, all modifications have been marked in the manuscript and uploaded as a supplementary file. ***Line numbers apply to document when "all markup" is selected.***

Reviewer #1 comments and responses:

[Comments from editor's letter]

The paper entitled "Propelling sustainable energy: Multi-omics analysis of pennycress fatty acid elongase 1 knockout for biofuel production" describes the metabolic and physiological characterization of the fae1 mutant in *Thlaspi arvense L.* (field pennycress), a non-model plant with potential applications in the biofuel sector. This manuscript combines transcriptomics and metabolomics data from developing and mature seeds, focusing on fatty acid profiles. While the study is well-written, certain aspects require clarification before publication.

Given previously published papers on fae1 mutants (Jarvis et al., 2021; Batsale et al., 2021; Ozsayhan et al., 2017), the authors should emphasize and justify the novelty of their study.

Response: We appreciate the reviewer's feedback. To address the comment regarding the novelty of our study in the context of previously published works, we modified the introduction beginning on line 121 to more clearly emphasize and justify our study's unique contributions.

The research reveals that from 14 to 20 days after pollination, no significant quantitative differences in fatty acids were observed between WT and mutant developing embryos. However, in mature ones, both quantitative and qualitative differences were identified. Could you please explain these differences?

Response: Upon reviewing the comments, one of our collaborators suggested that we analyze the fatty acid content using a mole-based representation (nmol/embryo) in addition to the mass-based representation (µg/embryo) that we originally used. Following this suggestion, we conducted a further analysis of the data. The mole-based analysis revealed that while the fatty acid content differs when expressed in mass (µg/embryo), this is not observed when expressed in moles (nmol/embryo). This finding indicates that the total number of fatty acid molecules remains consistent between the WT and mutant, suggesting that the observed changes in fatty acid quantity (µg/embryo) is likely a reflection of shifts in fatty acid chain length and molecular weight. We have revised our results and discussion accordingly and modified lines 151, emphasizing that while the WT appears to have more fatty acids by mass, which is an important agronomic trait for oil yield, the number of fatty acid molecules is comparable between the WT and mutant. Additionally, we have added supplemental figure S3 displaying this.

The authors suggest that the reduction in triglycerides in mature embryos (not observed in developing embryos) results from acyltransferases' inability to handle the accumulation of medium-chain fatty acids. They support this conclusion by referencing Bates et al. (2014), which demonstrated inefficient incorporation of hydroxyl fatty acids. However, comparing these metabolic situations may be problematic, as Bates' study involved an unusual fatty acid as the acyltransferase substrate. As they mentioned, overexpression of key acyltransferase could clarify this point.

Response: Thank you for your feedback. We agree that the comparison to Bates et al. (2014) may not be entirely appropriate, given the unique nature of the hydroxyl fatty acid substrate used in that study. Accordingly, we have revised our discussion starting on line 347 to include the possibility that the observed reduction in triglyceride content in mature embryos could result from feedback inhibition of diacylglycerol acyltransferase (DGAT), likely triggered by the accumulation of oleic acid and other long-chain fatty acids. This hypothesis is supported by activity assays on *Brassica napus* DGAT1, which demonstrated that its N-terminal domain functions as a negative regulator of triglyceride biosynthesis (Caldo et al., 2017). Furthermore, long-chain acyl-CoAs have been implicated in the regulation of lipid metabolism in mammalian systems (Hu et al., 2005; Nagy et al., 2014).

Reviewer #2 comments and responses:

[Comments from editor’s letter]

Dear Authors,

This study provides detailed insights into the effect of *FAE1* mutants on fatty acid (FA) composition and their impact on improving the cold-flow properties of the oil for sustainable aviation. The use of multi-omics approaches, including metabolomic and transcriptomic datasets on developing embryos, is particularly interesting. Additionally, the observation that *fae* mutants upregulated heat shock proteins is noteworthy. Experiments exploring the role of very long-chain fatty acids (VLCFAs) in heat tolerance using fae mutants would strengthen the paper, as would further investigation into cold tolerance, given that the mutants germinated well and earlier compared to wild types.

Response: Thank you for your kind comments on the multi-omics findings in our study. We also appreciate your suggestion to explore the role of VLCFAs in cold and heat tolerance. It is worth acknowledging that altering degree of unsaturation or chain length of fatty acids can influence membrane fluidity and the extent of cellular damage caused by temperature stress (Somerville and Browse, 1991; Iba, 2002). For instance, in tomato, *fad* overexpression mutants with increased levels of unsaturated fatty acids demonstrated enhanced cold tolerance (Domínguez et al., 2010). Furthermore, in cotton, *kcs13* overexpression mutants exhibited heightened sensitivity to cold stress (Wang et al., 2020). However, in this study *FAE1* encoding KCS18, is exclusively expressed in embryos and silicles (Joubès et al., 2008). As a result, whole-plant stress experiments on *fae1* mutants are unlikely to reveal shifts in heat and cold tolerance as the fatty

acid profiles of root, stem, and leaf tissues remain unchanged in *fae1* mutants. I have emphasized the embryo specific expression of *FAE1* in the discussion section of the revised manuscript, starting on line 311.

I have provided detailed comments in the attached version. The discussion section needs to be re-written. Much of it reiterates the introduction and results; instead, it should focus on the impact of the study and future directions. Therefore, I recommend a major revision.

Response: Thank you for your thorough review and suggestions. We agree that the discussion section needs refinement. Revisions of the discussion can be found on lines 281-301, 311-314, 328-335, 340-364, 366, 370, 376, 379, 381, 390, 399-413, 417, and 431-442.

[Comments made directly on manuscript]

Please write the full abbreviation when introducing it for the first time.

Response: The full abbreviation for *FAE1* was added to line 24.

Could you provide any evidence to support the claim that reduced erucic acid improves cold-flow properties?

Response: Thank you for your comment, we agree this point needs further clarification. The claim regarding the relationship between the length of fatty acids and cold-flow properties is supported by the findings of (Yang et al., 2018), which demonstrated that peanut and rapeseed biodiesel containing more short-chain fatty acid methyl esters (FAMEs) exhibited better cold-flow properties. Additionally, this relationship is further discussed in the following reviews: (Dwivedi and Sharma, 2014), Impact of cold flow properties of biodiesel on engine performance and (Leng et al., 2020), Cold flow properties of biodiesel and the improvement methods: A review. We have revised the manuscript on line 25 to replace "erucic acid" with "very long chain fatty acids (VLCFAs)"; erucic acid was originally highlighted due to its high abundance among VLCFAs. Additionally, the aforementioned citations were added to line 26.

Were the fae mutants evaluated for adaptation to cold or heat tolerance?

Response: As noted in our response above, *FAE1* has been shown in other species to be expressed in developing embryos and silicles, but not in vegetative tissues including leaves, stems, and roots. However, as part of a separate project characterizing multiple pennycress mutants involved in fatty acid biosynthesis, we have found that pennycress *fae1* mutants exhibited freeze and heat tolerance that is comparable to the WT. For heat stress experiments, we exposed *fae1* plants to elevated temperatures (28°C, 30°C, 32°C, and 34°C) at different growth stages, including flowering, and observed no differences in growth compared to the WT. Pollen

viability assessments revealed that *fae1* mutant pollen lost viability at elevated temperatures in the same proportions as the WT. Additionally, *fae1* mutants have been planted over several growing seasons in farm fields across more than six U.S. Midwestern states, ranging from Minnesota to southern Illinois (seeds sown in September or October and harvested in May or June). In these conditions, *fae1* plants grew, overwintered, flowered, and yielded similarly to the WT.

It would be better to include a pathway for better understanding.

Response: Thank you for this suggestion. We have generated a schematic diagram (Supplemental figure S1) showing how *FAE1* is involved in synthesizing erucic acid (22:1) from oleic acid (18:1), and how knockout of *FAE1* abolishes 20:1 and 22:1 production, resulting in increased 18:1, 18:2, and 18:3.

The data labels are hard to read. Could you modify this as a bar chart or find a better way to represent this diagram?

Response: Yes, the figure has been modified from pie charts to a bar chart. We believe this improves the clarity of figure 1C.

Please use color as it is hard to read the dotted labels for each tissue type.

Response: The greyscale that was previously utilized was changed to white, dark green, and light blue instead. This should help with the visual differentiation between the dots for the WT and two *fae1* lines (Figure 1A).

Write the labels directly in the diagram.

Response: Labels have been added directly into the diagrams for both Figures 1 and 2.

Are the colors in the bar chart color-blind friendly?

Response: Thank you for your comment. Yes, the colors in the bar chart are color-blind friendly. The BYU Colorblind Image Tester, a convolutional neural network trained on manually reviewed images, is 99.99% confident that this image is suitable for individuals with deuteranopia.

What are the time points? Please mention all: 14, 17, and 20.

Response: The time points are 14, 17, and 20 DAP. We have added these to the revised manuscript on line 257**.**

The same information is available in the introduction section. Consider reducing the text and jumping to the main discussion points right away.

Response: Thank you for your suggestion. We agree that the information is redundant, and we have reduced the text to minimize repetition. Lines 281-301 and 431-442 were deleted.

This was discussed in the results section. Please discuss the implications of your findings here.

Response: As addressed in our response above, the discussion has been extensively modified to better highlight the implications of our findings. The revised version now focuses on the novelty and implications of the study. We believe this improves the flow of the manuscript.

What might be the reasons for this observation?

Response: Thank you for your question. *FAE1*, which encodes β-ketoacyl-CoA synthase (KCS), plays a key role in the production of very long chain fatty acids (VLCFAs) such as erucic acid (C22:1). In *Brassica napus*, erucic acid is synthesized from oleic acid (C18:1) through two rounds of elongation catalyzed by the fatty acid elongation (FAE) complex. This complex consists of four ER membrane-bound enzymes, with KCS as the first and rate-limiting enzyme in VLCFA synthesis (Ghanevati and Jaworski, 2002). Overexpression of *AtFAE1* increases the abundance of the KCS protein, thus increasing the conversion of oleic acid to erucic acid and resulting in a higher abundance of VLCFAs.

We highly edited this portion of the discussion and this observation was removed as it was no longer relevant to the discussion (Lines 297-298).

So, VLCFAs are essential for high-temperature tolerance (e.g., germination), but not for cold? The mutants did not produce VLCFAs correct? Their FA composition mainly consists of mono- and poly-unsaturated FAs (long chains), right?

Response: Yes, this is correct. VLCFAs appear to be beneficial for high-temperature tolerance, as evidenced by the faster germination of the WT at elevated temperatures. This suggests that VLCFAs play a critical role in enabling the embryos to withstand heat stress. The mutants, which lack VLCFAs exhibited faster germination under cold conditions. This observation implies that the long-chain mono- and poly-unsaturated fatty acids, which are indeed more abundant in the *fae1* mutant, likely contribute to cold tolerance. We have emphasized this starting on line 330.

Mention the full abbreviation.

Response: The full abbreviation of TG was added to line 344.

What are all the ways to prevent starch accumulation? Discuss them in details here.

Response: Thank you for your suggestion. We have modified the discussion to include a paragraph about preventing starch accumulation starting on line 399.

Full abbreviation needed.

Response: The full abbreviation of FAMEs was added to line 535.

15-Nov-2024
Dr. Ana Paula Alonso
University of North Texas
BioDiscovery Institute
Denton, Texas 76201

MSID: PP2024-RA-02686D
MS TITLE: Propelling sustainable energy: Multi-omics analysis of pennycress fatty acid elongase 1 knockout for biofuel production

Dear Dr. Ana Paula Alonso:

I am pleased to inform you that your revised manuscript "Propelling sustainable energy: Multi-omics analysis of pennycress fatty acid elongase 1 knockout for biofuel production" has been accepted for publication in Plant Physiology. Your manuscript will be checked for consistency with journal formatting and image standards by one of our Science Editors, who will contact you shortly. They may have a number of queries and suggestions for you. Please keep in mind that they work to increase the online visibility of your article and its attraction for readers from the widest of backgrounds. You will have the opportunity to review any revisions, and I hope you will work constructively with them to get the most out of your research. You will also have the opportunity to review all changes when you receive the author proofs for your article.

Once validated for publication, your article will be sent to a Science Editor for a final review of your manuscript and source files. After your final files are uploaded, your article will appear in the next available issue and the online Preview version of your article will be posted after your signed Copyright and Checklist forms and final source files are received.

Please be sure to include the following in your manuscript file:
Harvard style citations
List of all author contributions and funding information at the beginning of manuscript after the titles and author list.
List all supplemental materials associated with the article, with titles, at the end of the ms, before the references and acknowledgments section.
Complete Figure Legends for all figures appearing in the article listed before the Literature Cited.

LICENSE INFORMATION Shortly after your paper is sent to production, you will receive an email from our publisher's author support team, **SciPris**, with information on article processing charges, as well as the types of Open Access license you would be able to purchase. The accepted version of your manuscript will not appear on our Advance Articles page until the license is selected and signed, so please look out for an email from SciPris. For more information see the Article Publication Fees section of our author guidelines: https://academic.oup.com/plcell/pages/General-Instructions

Finally, we encourage your submission of artwork for the journal cover. If there is an image or illustration related to your paper that you would like to have considered, please email it in .pdf or .tif format to clowe@aspb.org. An image title, brief, 2-3 sentence description of the image, and the name of the person credited for the image are also encouraged. If your image is selected to appear on the online cover of the journal, you will be contacted with the scheduled issue date and further information.

Thank you and congratulations on your Plant Physiology paper!

Sincerely,
Editor, Plant Physiology

=====
FIRST AUTHOR PROFILES
=====

We recognize the hard work that goes into being First Author of a Plant Physiology paper by publishing a "First Author Feature" for each First Author alongside your paper.

If the first author(s) would like to submit information for a feature on Facebook and Twitter, fill out the form by clicking on the link below:

Plant Physiology First Author Feature Form: https://forms.office.com/r/ZBCGZfhm8F
If you cannot access the form through the link above, please contact Rachel at rbelsky@aspb.org.

To upload an image of yourself to accompany your profile, please put your photo in the correct journal folder at the link below. You must label your image: Author First Name Last Name Article Title.

First Author Profile Image Dropbox: https://www.dropbox.com/scl/fo/xz029p7qjr2jml2rp2kmq/h?dl=0&rlkey=6fkxcaqmxx35ap152b2osy1fz

=====
IMPORTANT REMINDER: PEER REVIEW REPORTS
=====

If you opted to publish a peer review report along with your article during the original submission process, it will be prepared by the editorial staff and publicly posted with your manuscript, inside the zip file that contains any other supplemental material. As a reminder, the peer review report is a public record of all comments from editors and reviewers, as well as your prior responses, as you received them in the decision letters for each draft of your manuscript. If you agreed to publish this report and have changed your mind, or are not sure if you selected this option, please contact the editorial office as soon as possible before signing the license agreement from our publisher.

-- Reviewer comments:
Reviewer #1 (Comments for the Author):

I would like to thank the authors for taking my comments and suggestions into account. The revised article, which I believe has been significantly improved, addresses my initial questions about the text.

Reviewer #2 (Comments for the Author):

The authors have addressed all my queries and comments. I recommend this manuscript for publication in its present form.
